# COVID-19: through the eyes through the front line, an international perspective

**DOI:** 10.1186/s13756-020-00850-2

**Published:** 2020-11-07

**Authors:** Kevin T. Kavanagh, Judith Pare, Christine Pontus

**Affiliations:** 1 Health Watch USA, Somerset, KY USA; 2 Division of Nursing, Massachusetts Nurses Association, Boston, MA USA; 3 Health and Safety, Massachusetts Nurses Association, Boston, MA USA

**Keywords:** COVID-19, SARS-CoV-2, United States, Misinformation, Worker safety, Disparities, Personal rights, PPE, Personnel protection equipment, Public health, Masks, Lock downs, Centralized control, Strategic stockpile, Preparation, Spain, Singapore, Netherlands

## Abstract

COVID-19 is continuing to ravage the globe. In many Western Countries, the populous has not embraced public health advice which has resulted in a resurgence of the COVID-19 virus. In the United States, there is an absence of a coordinated Federal response. Instead, frontline workers and average citizens are having to cope with extensive mixed messaging regarding mask usage and social distancing from the highest levels of government. This has resulted in the United States not being able to achieve a low level of infection since the pandemic began. In addition, many citizens hold a profound belief that individual freedoms must be preserved, even at the expense of public health; and view the wearing of masks as renouncing this right. These engrained political beliefs can be traced back to the late 1800s. The response of the United States has also been hampered by a highly cost-efficient healthcare system, which does not provide universal care and has a just-in-time supply chain, with far too few supplies in reserve. This efficiency prevented a rapid scaling up of the healthcare response, which resulted in severe deficiencies in available personal protective equipment (PPE) and healthcare staff. To compound issues many healthcare staff are not provided an economic or healthcare safety net. Other frontline workers, such as those who work in transportation and food services, are working under even greater adversities. Many of these workers are from diverse backgrounds, who, along with their families, are at even greater risk for COVID-19. This vulnerable population of frontline workers are faced with a choice of going to work with inadequate PPE or placing food on their families’ table. In the United States, official recommendations seem to be ever changing, based more upon supply and test availability, than on science. We must rely on science and learn from the lessons of past pandemics or we will relive, even to a greater degree, the deaths and devastations experienced by our ancestors over 100 years ago.

## Introduction

COVID-19 has devastated the World, affecting every country in its path. The 2020 COVID-19 Conference “Though the Eyes of the Front Line” had as its underlying goal, to educate nurses, health care professionals and the general populace about the challenges that this global pandemic has caused. Additionally, the conference focused on the consequences of the outcomes of COVID-19 which spread globally including the staggering loss of lives that continue to generate from the lack of leadership, transparency, and trust in the science to combat the disease.

Much of the world has been surprised at the difficulties the United States is having containing this virus. Two major factors came into play. The first is a belief it could not happen in the United States; epidemics will happen only in underdeveloped countries with a rudimentary public healthcare system.

Secondly, the view that individual freedoms and the rights of local governments over federal governments has provided an added barrier in controlling the spread of the COVID-19 across our nation. American Medical Association President Hunter Maguire [1] stated, “A difficulty in dealing with infectious diseases in America was the rooted dislike to the curtailment of the personal liberty of the citizen for the benefit of the people at large.” Maguire, then described the need for an active coordinated Federal response. Tellingly, this speech was given in 1893 and not much has changed. Americans still hold individual freedoms as a vital foundation for its democracy. The consequences of the United States uncoordinated federal response is evident in the statistical data compiled by Johns Hopkins [2], which has to date resulted in more than 9,220,933 persons diagnosed with COVID-19 and over 230,000 deaths across the more than 50 states and territories.

The United States’ historical values and culture has been ideal for confronting almost insurmountable challenges in the past but for controlling a viral pandemic they are counterproductive. One only needs to look at the events that began in February, 1918 when what was thought to be “severe influenza” was initially identified at Camp Funston, in Haskell County Kansas. Barry [3], stated that at first it seemed like nothing to worry about, certainly nothing as serious as the measles outbreak with its dire complications. However, that was the beginning of multiple waves of the virus that would claim more than 50 million lives worldwide and an estimated 675,000 Americans died after exposure to the virus [4]. During the four and one-half years of battling the virus, the responses of the public health officials evidenced a new allegiance to science, and this sparked the development of new theories of disease prevention, diagnostics, and treatments. This allegiance was tested and, in many ways, fractured when COVID-19 emerged during the winter of 2020 and concerns of a new pandemic were viewed by some as “fake news”.

The belief in invincibility and that a pandemic could never reach the United States (U.S.) led to a depletion of personal protective equipment (PPE), including N-95 masks in the U.S. stockpile. The federal stockpile has been depleted for over a decade. Even though the original projected need for an epidemic response in the United States was 3.5 billion N-95 respirators, the stockpile was only designed to hold 85 million masks, with only 12 million actually present. The remainder were largely depleted during the N1H1 pandemic [5]. The Washington Post reported that the reserves of N-95 respirator masks were not “significantly restored” after millions of masks were distributed in response to the H1N1 influenza pandemic from the stockpile in 2009. Consequently, the U.S. supply chain is in shambles with many facilities still having to deal with a just-in-time 3 to 5 days’ supply of personal protective equipment (PPE).

The dysfunction of the United States’ Federal Emergency Management Agency (FEMA) became evident during Hurricane Katrina in 2005, and was exacerbated by the dismantling of the United States’ pandemic response team in the spring of 2018. All of these take-aways went largely unnoticed by the American people, leaving few protections and a lack of understanding and confusion about the role of leadership. Mixed messages, from leaders, seemingly driven by a willingness to not fully disclose the poor performance of our healthcare system and the early rapid spread of the virus. Public communications regarding the need for masks, along with the dangers of the virus were both conflicting and confusing, [6] with messengers repeatedly changing their stances. There was expressed concern by some leaders to not cause the public to panic [7]. This also led to public confusion and conspiracy theories as to why stringent advisements were being made if the virus was not that dangerous. Hospitals and health care settings became environments that served as breeding grounds for workers who, because they were not given adequate protection became possible sources of spread for COVID-19 [8].

According to the Food and Drug Administration (FDA) [9] medical personnel wear respirators because they “filter out at least 95% of airborne particles”, and this is an essential health and safety requirement. Along with serious shortages of PPE, the supply of ventilators and hospitals beds were gravely lacking in the U.S. The healthcare system in the U.S. ran in crisis mode with shortages of staff, equipment, training confusion and misdirection.

This is in contrast to Southeast Asian countries who learned from the past epidemics of MERS and SARS.

## Progress from other nations

Singapore was one of the hardest hit countries with SARS and prior to the SARS-CoV-2 pandemic had almost three N95 masks in their stockpile per citizen. A lesson can be learned from Singapore regarding the need for Universal Health Care for all residents. Singapore’s fourth wave of infections came from migrant workers who were housed up to 15 to 20 workers in each bedroom, and many of whom encountered barriers in healthcare access [10]. The countries in Southeast Asia were more than prepared with infrastructure preparations, including ready access to medical records and cell phone identification of contacts. In the United States, the lack of standardization and the timely communication of medical records information along with the health disparities that existed prior to the pandemic have become more evident and continue to contribute to increased inequities experienced by the nation’s most vulnerable populations.

A number of different strategies were used by other countries around the world to crush the pandemic curve. In the Netherlands, [11] there was not an emphasis on wearing masks, but there was almost uniform public acceptance to a lockdown which resulted in almost vacant streets in cities and allowed for effective case tracking. Similar to the United States, half of the deaths occurred in Nursing Homes and Long-Term Care Facilities, necessitating prohibition of visitors.

Spain’s Government is similar to the United States in that it has strong local control. Initially, Spain did not have uniform or nationwide recommendations. However, on March 15th, as the pandemic started to progress, the central government took control of all measures and locked down the entire country [12]. Unlike the United States, Spain’s national compliance with their lockdown was very high. In some regions of Spain, the wearing of masks was compulsory. Universal masking is required in hospitals, seroprevalence of SARS-CoV-2 in healthcare workers was twice that of the general populations, 10% versus 5.2% respectively.

Unfortunately, learning from past mistakes and from others appears to be hard. In much of Europe, a second wave is occurring as a large portion of their populous has become weary of public health measures and appear to be adopting the laissez faire attitude and lifestyles of those denying the lethality of the coronavirus [13]. In the United States, we still have not yet learned from others and despite the experiences in other nations, many still believe this is ‘political’ and not a real concern.

## The Commonwealth of Massachusetts

In Massachusetts, healthcare facilities were the most common workplaces to receive complaints accounting for 30% of the total. Examples included reuse of N-95 masks and contaminated gowns, not having ready access to disinfectant wipes and minimal availability of PPE; and According to Jodi Sugarman-Brozan, Executive Director, Massachusetts Coalition for Occupational Safety and Health (MassCosh), “Most if not all of these complaints were closed by Occupational Safety and Health Administration (OSHA) without even an investigation.” [14].

Sugarman-Brozan described the inadequacy of sick leave and workers’ compensation benefits. This lack of a healthcare and economic safety net for workers is a concern because it places a sick worker in the position of choosing between going back to work or paying for rent and placing food on one’s family’s table.

In Massachusetts, the SARS-CoV-2 positivity rate for Black and Hispanic residents is three times that of white residents [14]. They are also play a disproportionate role in our front-line response. Black workers comprise 33.3% of taxi and ride service drivers, 17.9% of bus drivers, 28.9% of personal care attendants, 41.2% of nursing and home care aids, and 37.0% of Licensed Practical Nurses [14]. Hispanic workers comprise 79.5% of laundry and dry clearing workers, 33.9% of food preparation workers, 23.8% of childcare workers, 28.9% of personal care attendants, 19.9% of nursing and home care aids, and 31.5% of Janitors and cleaners [14]. Not only are these groups overrepresented in front-line workers but when infected, the workers and their families also have decreased access to healthcare.

Finally, no one is counting fallen and afflicted healthcare workers. The Centers for Disease Control and Prevention’s (CDC) COVID-19 Data tracker has data from over 5 million individuals, but healthcare personnel status was only available for 22.8%. The only data available was recovered versus death status which represent 73.5% of known healthcare workers [15]. The CDC has reported over 786 deaths in 201,992 healthcare workers with COVID-19. But because of the lack of complete data, healthcare worker deaths as of Oct. 7, 2020 may be over  4500. And this is not counting the number of workers stricken with lasting disability due to heart and pulmonary disease that is an outcome of the virus.

## Summary

One of the most important lessons this pandemic has taught us is that access to healthcare is needed for all. Infectious disease does not respect socioeconomic or geographic boundaries. It was evident that there were several different strategies which could be successful in controlling viral spread. However, the overriding factor in success was the widespread acceptance by the populous of public health control measures. The disparities that existed prior to the pandemic has become more evident and contributed to increased inequity among the nation’s most vulnerable population. The uninsured and homeless in the United States are potential foci for reinfection of our entire population.

In addition, the United States needs to focus attention to all frontline workers. During the 2020 conference, there were stories of lack of appropriate PPE and changing recommendations. This was especially prevalent in the guidance for the type and need for masks. Recommendations appeared to be more based upon availability, rather than worker protections and public safety. With the recent revelations on the aerosolization of the virus, [16] all frontline workers should, at the minimum, have access to goggles, and a new N95 mask for each shift, and more stringent measures for staff working with COVID-19 positive patients. PPE shortages are still existing in the U.S. at the time of this writing.

According to a report in the Morbidity and Mortality Weekly Report of the U.S. Centers for Disease Control and Prevention, (2020) [17], more than 3000 frontline workers in 12 states, roughly 1 in 20 had antibody evidence of a previous COVID-19 infection, but 69% of those infections had never been diagnosed.

Kaiser Healthcare News reported that between March and June 30th, 2020 there were over 4100 healthcare worker complaints in the United States regarding protective gear [18].

During a webinar hosted by the Society of Critical Care Medicine, Anthony Fauci, MD, director of the National Institute of Allergy and Infectious Diseases, stated, “It is now clear that about 40–45% of infections are asymptomatic.” Asymptomatic carriers may be responsible for up to 50% of SARS-CoV-2 acquisitions [19].

The response of the current public health officials and governmental leaders must now reflect the lessons that were learned but apparently forgotten following the 1918 pandemic. An allegiance to science is necessary in order to eradicate the destruction of COVID-19. If these lessons are not learned, we are destined to double the death and devastation experienced by our ancestors 100-years ago.

## Data Availability

Not applicable.

## Abbreviations

PPE: Personal Protective Equipment
FEMA: Federal Emergency Management Agency
FDA: Food and Drug Administration
CDC: Centers for Disease Control and Prevention
